# Guidelines for open peer review implementation

**DOI:** 10.1186/s41073-019-0063-9

**Published:** 2019-02-27

**Authors:** Tony Ross-Hellauer, Edit Görögh

**Affiliations:** 1 Know-Center GmbH and Graz University of Technology, Inffeldgasse 13, 8010 Graz, Austria; 2 State and University Library Goettingen, Goettingen, Germany

**Keywords:** Peer review, Guidelines, Open peer review, Scholarly publishing, Open science

## Abstract

Open peer review (OPR) is moving into the mainstream, but it is often poorly understood and surveys of researcher attitudes show important barriers to implementation. As more journals move to implement and experiment with the myriad of innovations covered by this term, there is a clear need for best practice guidelines to guide implementation. This brief article aims to address this knowledge gap, reporting work based on an interactive stakeholder workshop to create best-practice guidelines for editors and journals who wish to transition to OPR. Although the advice is aimed mainly at editors and publishers of scientific journals, since this is the area in which OPR is at its most mature, many of the principles may also be applicable for the implementation of OPR in other areas (e.g., books, conference submissions).

**Electronic supplementary material:**

The online version of this article (10.1186/s41073-019-0063-9) contains supplementary material, which is available to authorized users.

## Background

Openness in peer review has been labelled an ‘increasing trend’ [1]. Although it has been a feature of publishers such as *BMJ* and *BMC* (formerly *BioMed Central*) for almost 20 years, it has gained ground recently, spurred by the *Open Science* agenda for increased transparency and participation in scientific processes. Many publishers and journals already run some form of open peer review, including *BMC* (owned by *Springer Nature*), *BMJ*, *Copernicus*, *eLife*, *EMBO Press*, *F1000Research*, *Nature Communications*, *Royal Society Open Science* and *PeerJ*. In 2018, an open letter was published in Nature [2] calling for publishers to begin to publish peer review reports. Editors and publishers representing over 100 journals have so far signed to acknowledge that they have either already implemented, or plan to implement, the publication of peer review reports.

Open peer review (henceforth OPR) can take place in different stages of the review process—pre- or post-publication—offering extended communication and knowledge exchange between researchers. Platforms and publishers implement OPR tools to encourage wider and more transparent discourse within the review process. Yet, the openness of these systems often differs in terms of what is revealed to whom and when. To bring clarity to how the term ‘open peer review’ is used, a systematic analysis by one of the current authors analysed definitions of OPR in the literature, identifying seven core traits which were used in 22 distinct configurations [3]. Across all definitions, the main core elements were revealing reviewer identities (open identities) and publishing reviews (open reports).

Attitudes to the elements of OPR vary greatly among researchers. A recent study by one of the current authors [4] found the majority of respondents to be in favour of open peer review becoming mainstream scholarly practice, with high levels of support for most traits of OPR, particularly open interaction, open reports and final version commenting (although not for opening reviewer identities to authors). Other studies also report a generally positive attitude towards open peer review [5, 6]. Opening up identities and reports are presented similarly as primary issues in numerous studies. Baggs et al.’s analysis of nursing journals in 2008 framed transparency and revealing reviewer identities as holding the potential to increase professionalism, communication, accountability and fairness. However, the majority of respondents in the study preferred closed identities in the review process to avoid interpersonal conflict and political issues [7]. Although a very recent study on the global state of peer review by Publons reinforces these findings, it suggests that attitudes towards OPR are shifting as the new generation of researchers is more likely to review for journals with OPR options [6]. Furthermore, publishing channels with clear open access and open peer review policies are becoming a major factor in choosing dissemination tools or services. A recent study of *F1000Research* authors rated transparency of the peer review system, including revealing reviewer’s names, as important or very important reasons for publishing with that venue by the majority of respondents [5].

OPR is attracting increasing attention, but there are a diverse cluster of interrelated but distinct innovations, which can be combined in a myriad of combinations, that fall under this term. Hence, any publisher wishing to move in this direction faces crucial choices about which elements of openness to embrace, and these decisions will in their turn expose them to potential advantages and disadvantages for the quality of their peer review systems. Which OPR system is optimal, for which communities and in which circumstances? How should these systems be implemented, and what opportunities and pitfalls should be recognised? Given such growing interest in open peer review, combined with such a proliferation of options for *how* to ‘open’ peer review, it becomes urgent to offer clear guidelines for those publishers and editors interested in taking up such practices.

This article addresses this need. It presents a series of structured guidelines for the introduction of the various open peer review traits for publishers and editors. The guidelines were produced in close collaboration with a group of experts in peer review, and especially open peer review, from publishing and research. Methods included via background research, expert interviews and an expert synthesis/validation workshop. The guidelines seek to be of use to those who oversee the peer review of manuscripts for publication who are considering introducing more transparency or inclusivity to their peer review processes by implementing any of the innovations grouped under the term ‘open peer review’. The paper first gives general advice which cuts across all OPR elements, before going on to detail specific advice for each of the OPR elements. Although the advice is directed mainly at editors and publishers of scientific journals, since this is the area in which OPR is at its most mature, many of the principles may also be applicable for the implementation of OPR in other areas (e.g., books, conference submissions).

## What issues with peer review does open peer review address?

Peer review is a method of scholarly quality assurance that serves to validate the soundness, substance and originality of a work, to assess and help improve it until it meets required standards for these criteria, as well as sometimes to select for ‘appropriateness’ or ‘fit’ for certain venues. Peer review, as it relates to scholarly publishing, is here understood as the formal scholarly process where an editor sends copies of a manuscript to neutral third-parties judged knowledgeable enough to be able to comment on its quality and suitability for publication. Standard peer review is understood to be typically:

- *Anonymous*: either reviewer identities are kept from authors (single-blind) or author and reviewer are unknown to each other (double-blind). In some cases, authors identities are also hidden from editors (triple-blind)
- *Confidential*: the process takes place behind closed doors (or, rather, password privileges) and reviews are not published
- *Selective*: reviewers are chosen by the editor

As has been said, open peer review is a complex phenomenon, which actually represents a range of possible innovations to standard peer review processes. Each of these innovations addresses different issues with standard peer review. Following the summary presented in [3], we offer the following, non-systematic, review of the following issues that the various traits of open peer review are proposed to address:

- *Accountability*: The increased transparency offered by open identities and reports could increase accountability and make reviewer conflicts of interest more apparent. Open participation could reduce possible problems with biases or elitism associated with editorial selection of reviewers [8]; on the other hand, it could facilitate engagement by those with conflicts of interest (particularly where anonymity is allowed). Open identities are sometimes theorised to discourage reviewers from making strong criticisms, especially against higher-status colleagues—if true (and there is little evidence against which to judge this), this could subvert review by weakening criticism [3].
- *Bias*: Open reports allow the scientific community to examine how publication decisions were made. However, open identities remove the anonymity for reviewers (single-blind) or authors and reviewers (double-blind) which has traditionally been used to counteract such biases [9, 10].
- *Inconsistency*: Open identities and open reports could improve the quality of reviews, encouraging reviewers to be more thorough in their assessments (although there is too little evidence to say if this is the case) [11]. Open participation, by increasing the number of potential reviewers, could lead to more thorough review processes [12] (although note that open participation processes often fail to attract large numbers of comments). Some evidence suggests open interaction could increase the accuracy of reviews [13].
- *Time*: Peer review often takes a long time. Publishing manuscripts online in advance of peer review, either as pre-prints or as part of the publisher workflow, speeds up dissemination and (in disciplines like Physics) enables researchers to claim priority in a finding [14]. Open platforms could help avoid cycles of review, where articles are submitted to various journals before finally being published and are reviewed anew each time. However, open identities and open reports could increase delays by increasing the number of reviewer invitations needed to secure the required number [11], and open interaction could delay processes by leading to cycles of comments back and forth between reviewers and authors [15].
- *Incentive*: If review reports were published alongside reviewer names, it would be easier for researchers to claim credit for these activities, thus incentivising review [16]. Open participation could incentivise researchers by allowing them to seek out works they want to review.
- *Wasted effort*: Rather than hiding the useful contextual information contained in peer review, open reports would make this available [17].

## Methods

The guidelines were created in close consultation with a group of experts. The views of expert participants were sought via an interactive meeting, a brief pre-meeting questionnaire and subsequent sharing of drafts of the guidelines with all participants for feedback. Participants were chosen for their expertise in peer review—especially open peer review—and, although care was taken to include conservative voices, the topic and aim of the meeting attracted people ‘open’ to open peer review. The aim was to have most large publishers represented. The list of 15 experts who took part in the workshop, and others who contributed to the pre-meeting questionnaire and commented on drafts of the guidelines, is listed in the ‘Acknowledgements’ section of this article. All consented to be named. They represent experts in peer review and open peer review from many major publishers (*BMC* (part of *Springer Nature*), *BMJ*, *Copernicus Publications*, *eLife*, *Elsevier*, *F1000Research*, *Hindawi*, *MDPI*, *Nature* (part of *Springer Nature*), *PLOS*, *Royal Society Open Science*, *Taylor & Francis*, *Wiley*), along with representatives from Publons and the PEERE research consortium. All data was collected and analysed by the current authors: Dr. Tony Ross-Hellauer (male) and Dr. Edit Görögh (female), researchers in *Open Science*. The authors have rich previous experience in performing qualitative analysis in these areas. Seven of the participants were already known to the authors, but otherwise, no relationships were established prior to study commencement. Prior familiarity with some participants means, however, that the authors were to varying degrees aware of the broad stance of some participants towards open peer review before the study commencement.

### Scoping interviews

Initial scoping of the issues was conducted via three initial semi-structured interviews with academic publishing professionals whose publishing portfolios included open peer review journals. Participants for this stage were chosen for their familiarity with the theme. All those approached assented to interview. Interviews were conducted by the current authors between 5 and 18 December 2017 via Skype. No audio or video recordings were made, but detailed notes of participant answers were taken by the interviewers. Each interview lasted between 30 and 60 min. Notes were then shared with participants for any post-interview comments, corrections or additions.

### Pre-meeting questionnaire

A week in advance of the workshop, a short online pre-meeting questionnaire was distributed to workshop attendees via Google Forms, consisting of three open questions: ‘If a publisher or journal is interested in implementing OPR in some form, what advice would you give them on how to get started?’, ‘What opportunities should they look out for?’ and ‘What pitfalls should they look out for?’. In total, 14 responses were received between 16 and 23 March 2018. No personal data save names and email addresses were collected. All data were kept and stored in accordance with data protection regulations. The authors of this study then collaboratively coded answers to the three open questions, iteratively grouping them using a grounded theory approach. This resulted in a list of preliminary common categories and themes, which were then used as the basis for further investigation at the interactive workshop: technological/process issues, engaging/listening to communities, being pragmatic—where to get started, biggest drivers for selling concept, biggest problems to watch out for, how to set goals and evaluate performance.

### Interactive workshop

The interactive workshop took place on 27 March 2018 at *Springer Nature*’s Stables venue in London, UK. Participants were identified via purposive sampling, with the aim to include representatives of most of the major publishers who run or are experimenting with open peer review processes, but also include publishers with more traditional peer review systems, as well as funders and researchers where they had previously demonstrated interest in these issues. Twenty invitations were issued via email, of which two were declined (for lack of time) and two received no response. One confirmed participant was unable to attend due to illness, meaning that 15 participants took part in the workshop (eight male and seven female). All represented major publishers except one participant from Publons (a peer review analytics company) and one active peer review researcher (from the PEERE network).

The meeting took the form of an interactive workshop which lasted 90 min. No audio or video recording of the workshop was made, but extensive field notes were kept by the researchers. No one else was present besides the participants and researchers. The session was moderated by Edit Gorogh with note-taking by Tony Ross-Hellauer and additional facilitation from Elisabeth Moylan, one of the attendees. Edit Gorogh first presented the motivation for the exercise. Participants were first asked to consider in pairs to consider the questions: ‘For those who have implemented/experimented with OPR, what one thing would you have done differently?’ or ‘For those without OPR experience, what’s the one thing you’d really like to know first?’ Each pair was then asked to report back to the group the main points of their discussion, enabling free-form discussion of main points of interest.

The group was then split into two, and each group asked to discuss and record their main advice under each of the headings identified as main common themes following the interviews and questionnaire. Following group discussion of 15 min, each group reported back to the whole group, whereupon each group could give feedback on each other’s answers. In a final stage, the group was split into four sub-groups (self-selected) who then each provided advice specific to individual OPR traits (open identities, open reports, open interaction, open participation/pre-review manuscripts). Again, each group reported back to the whole group for further discussion. The researchers kept notes during the discussion of major and minor themes.

### Iterative drafting of guidelines text

Following the workshop, the authors of this study used the answers from the workshop to further refine the structure and content of the guidelines. First, notes taken during the meeting were collated, and major and minor themes identified according to a grounded theory approach employing open and axial coding. Following this, a first draft of the guidelines was written and shared with all expert participants via Google Docs for collaborative feedback and further refinement. Following submission of this manuscript, and resulting useful critical comments from two very thorough reviewers, a revised draft was created in which the advice was further refined. This version was then shared with all participants in December 2018 for their further feedback before resubmission.

## General advice on implementing open peer review (OPR)

### A) Set your open peer review goal(s)

#### A1. Decide what you would like to achieve with OPR

Any journal editor or publisher wishing to implement some form of OPR would be well-advised to first do their homework. What do you want to achieve? How? For which reasons? Answering these questions first will enable you to orient your engagement with OPR. Examine which particular aspects of your peer review processes you would like to improve. For example, do you want to increase the transparency of your processes, give credit to peer reviewers, enable greater participation, or just speed up the peer review process? Being clear on these primary goals is vital.

#### A2. Acquaint yourself with the differences between the elements of OPR

As discussed above, ‘open peer review’ can mean different things to different people. As a first step, familiarise yourself with the differences between each of these elements. For example, one of the current authors created a taxonomy of seven core traits:

1. Open identities: authors and reviewers are aware of each other’s identity.
2. Open reports: review reports are published alongside the relevant article.
3. Open participation: the wider community are able to contribute to the review process.
4. Open interaction: direct reciprocal discussion between author(s) and reviewers, and/or between reviewers, is allowed and encouraged.
5. Open pre-review manuscripts: manuscripts are made immediately available (e.g., via preprint servers like arXiv) in advance of formal peer review procedures.
6. Open final version commenting: review or commenting on final ‘version of record’ publications.
7. Open platforms (‘decoupled review’): review is facilitated by a different organisational entity than the venue of publication.

Read widely to familiarise yourself with the pros and cons of each of these elements (a primer with links to some literature is given above in the ‘What issues with peer review does open peer review address?’ section and a longer list of secondary reading is included in the Additional file 1).

#### A3. Decide which elements you would like to implement

Being clear on your primary goals and relating them to specific elements of OPR will enable you to begin to build a provisional strategic plan for OPR implementation. Further refine this by studying existing models and OPR implementations through publisher websites, published literature, presentations and online resources. Use industry contacts and discussion platforms to discuss and learn lessons from publishers and journals who have already implemented OPR procedures. Be aware that the resource and time commitment is dependent on the elements selected—disclosing names of reviewers is relatively straightforward whereas publishing a full peer review history for each paper requires significant investment (see also the ‘Assess technological feasibility of various options’ and ‘Assess the costs of various options’ sections below).

### B) Listen to research communities

#### B1. Be conscious of, and sensitive to, community differences

Be conscious that there will be differences in perceptions and willingness among different research communities. For instance, some disciplines have more of a tradition with double- or even triple-blind review and this might lead to more resistance to openness in peer review. If you are a publisher overseeing peer review at many journals, consider starting with particular disciplines that are more open to trial OPR, especially those where other journals in the field already use OPR (although note that this may be challenging for broad scope interdisciplinary journals).

#### B2. Consider surveying community opinions

Consider directly surveying community opinions regarding open peer review models to gauge attitudes. This may work especially well for journals with close-knit communities—for example, society journals, which regularly seek feedback from authors or society members regarding journal policies. Alternatively, or as a complement to this strategy, consider targeted ‘qualitative interviews’ to gather insights from those with particularly strong opinions regarding open peer review.

#### B3. Communicate your goal with the stakeholders and research community

Engage journal communities—firstly by consulting your editorial board and reviewers to get them on board with the idea. It may be necessary to ‘sell’ the benefits of opening up peer review and provide reassurances. A committed and engaged Editor who can drive such discussions may help here. Find keen researchers to work with and gauge interest in the model among communities the journal serves. Let reviewers, authors and readers know in advance, and if you are unsure of how such developments might be received, consider announcing plans in a journal editorial and seeking community feedback. In any case, include requests for community feedback in any such announcements to ensure alignment with researcher attitudes.

### C) Plan technologies and costs

#### C1. Assess technological feasibility of various options

A deciding factor in your prioritising the elements of openness to include will be the technical possibilities of your system. Whether you are a small publisher using open source software or a large publisher which uses one of the major manuscript handling services, if your electronic editorial office and production/publication systems and workflows cannot currently be easily configured for OPR elements, they may be difficult and/or expensive to implement.

#### C2. Assess the costs of various options

It is important to recognise potential costs in advance. As things stand, there is a lack of infrastructure to facilitate automated workflows for many of the elements of OPR. Hence, development costs may be a major barrier—especially for smaller players. Ask yourself: Which options does your system already support, and do you have the technical staff or resources to fund system development? Consider also that costs will likely not only be in initial implementation (e.g., custom system development), but also ongoing support costs (e.g., staffing). If your needs would require significant custom system adaptations from a third-party service provider, you might consider partnering with other publishers who use these services to spread costs in implementing these changes. Alternatively, some platforms are now offering specific OPR functionality to work together with more traditional publishing services. In any case, be aware that there will usually be different ways, with differing levels of elegance and cost, to implement OPR options. For instance, publication of peer review information could be as simple as manually compiling review components and publishing a single document as Additional file 1 or as complex as an automated (XML) workflow where each element is published separately (see also the ‘Open reports’ section).

#### C3. Consider workaround options for piloting

If you are just experimenting with OPR, it may be that rather than immediately extending your whole publication architecture, it might be better to start small with workarounds, although be aware that ad hoc workarounds may produce a less smooth user-experience which could affect uptake and user attitudes to the experiments. Consider, however, that the sub-optimal nature of workaround solutions may then become an inhibiting factor in the success of the experiment. One solution here would be for a third-party OPR platform to offer their service as a plug-in to existing workflows for conducting such experiments.

### D) Be pragmatic in your approach

#### D1. Set priorities and consider a phased approach

Be flexible and choose your battles carefully. Change is difficult and you may run into problems if you try too many things at once. Your communities may be more receptive to some elements than others, and so, prioritising the areas you would like to change and being prepared to compromise from the ideal situation or at least take a phased approach may help you maintain traction and community buy-in. It will also make it easier to systematically assess the success or otherwise of any particular innovation.

#### D2. Consider making options optional or piloting them first

For elements you would like to introduce but think might prove controversial, you could make them optional. Thereby, it is possible to signal your support for this innovation while allowing reviewers or authors to opt-out. Note, however, that default policies may significantly affect outcomes—if the default policy is opt-in, this might lead to lower participation than if the default were to opt-out, for instance. If reactions among research communities may be uncertain, consider introducing OPR through a pilot study with an accompanying survey for participants which would show that any final decisions would be based upon real experiences, whilst allowing the journal to experiment with the confidence of the community.

### E) Further communicate the concept

#### E1. Engage the community, especially via ‘open champions’

Once you have decided on the model you’d like to move to, you have your communities on board, and have prioritised which OPR elements to implement, you will still need to sell your communities on the concept. As a general strategy, you should engage with the research community to find academics who are enthusiastic about OPR to be ‘open champions’ in advocating to their peers—for example, by engaging people who responded positively to your initial community consultation in step B. Moreover, the arguments above in favour of the various aspects of OPR will help sell the concept, especially with regard to increasing transparency, enhancing credit for review activities and demonstrating and (although this is an understudied area) potentially enhancing the quality of reviews.

#### E2. Be aware that communication is key and terminology is important

Misunderstandings could derail processes. As the stewards of the peer review process, publishers and editors have a duty of care to ensure reviewers and authors fully understand the systems of peer review in which they participate and its potential advantages and disadvantages. Use editorials, webinars, infographics and/or blog posts to articulate decisions and justify why these decisions have been made. Formulate clear policies which are easily findable on journal webpages for authors and reviewers.

### F) Evaluate performance

#### F1. Have a clear framework for assessing success

There is a need to track review quality and acceptance rates to monitor how OPR affects processes. As said above, it is good to decide a vision for the kind of peer review you want in the context of your end-to-end publication workflow and then prioritise goals in order to reach this vision. A key part of this planning should be deciding how you will define and evaluate success. Have a clear framework for assessing success (‘of what on whom’, so on specific measures and specific population clusters). Systematically collect data and study the impact of the practice on journal performance. Key questions could be the following: is review quality improved? Is it more difficult to find reviewers? Are review times impacted? Are open reports being consulted and re-used? It is also advisable to consult with your journal community once the new process has been in place for some time, perhaps via survey, to gauge the development of their attitudes towards processes. Important here is to establish ex ante which quantifiable measures or performance indicators will be used for internal analysis. Outcomes should always be considered on an appropriate time scale, however. Change takes time.

#### F2. Accept that change takes time, but adjust if necessary

Bear in mind that cultural change takes time, and so, even where uptake is not as quick as wished, the broader ethical aims of transparency and accountability in scholarly publishing might make persistence desirable in spite of low uptake. However, if things really are not working, then it may be necessary to re-evaluate your goals in light of lessons learned. For example, revisit the advice in the ‘Set priorities and consider a phased approach’ and ‘Consider making options optional or piloting them first’ sections to consider phasing individual elements or making them optional.

#### F3. Share your results with the community

Giving updates on progress will enable community engagement, keeping authors, reviewers, editors and publishing staff updated on the progress of your initiative. These updates will also help others decide whether and how to implement similar approached. There is currently a lack of real scientific evidence on the efficacy of many traits of OPR. Once enough evidence has been gathered, consider writing up the results as a scientific study for peer-reviewed publication. Alternatively, consider partnering with peer review researchers from the start to ensure data is well-formed for such analyses and to enable rigorous external scientific analysis.

## Advice on implementing specific elements of open peer review

### G) Open identities

Open identities peer review is a review where authors and reviewers are aware of each other’s identities. Reviews with open identities can seem to be more constructive in tone [18]. There has been some evidence [11, 19] that finding reviewers to review openly might be more difficult, although others have found no such negative consequences [18]. If you opt for open identities, however, it may be advisable to do the following:

#### G1. Devise strategies to compensate for the possibility that open identities might make it harder to find reviewers

Make sure to create a new standard reviewer invitation email which includes a clear description of the open identities review process and its potential advantages as well as disadvantages, as well as a standard follow-up text which goes deeper into these issues to convince those who are reticent. If you are keen to invite a specific person who is reticent, be ready to negotiate to persuade them by further explaining or, for example, offering more time to review if a reviewer believes they will need more time under such circumstances.

#### G2. Be alert to possible negative interactions and have a workflow for dealing with them

A common concern regarding open identities is that junior researchers who give negative reviews to more senior colleagues may face retaliation in some form. While it is important to note that there is at present only anecdotal evidence of this, such concerns seem nonetheless very common and underlie much of the negative response to open identities. It is hence essential to deal with these concerns early on to set authors and reviewers at ease and limit the risk to which researchers who take part in review processes are exposed. Have in place clear processes for dealing with any reviewer concerns and encourage any reviewers experiencing negative consequences to contact the journal as a matter of academic ethics. Publishers should also use their experiences to contribute to the evidence base on this issue by monitoring whether open identities leads to more positive reviews overall, for example.

#### G3. Enable credit

Wherever reviewer names are disclosed along with publication, be sure to use identifiers (e.g., ORCID) to link that activity to reviewer profiles and further enable credit and career evaluation. Assigning persistent identifiers like ORCID is a crucial element of best practice within current publishing practices. Moreover, if your journal is not yet participating with Publons, also consider enabling this partnership to further enable reviewer credit.

#### G4. Consider piloting or making open identities optional

This point was made above for OPR elements in general, but is worth applying to open identities specifically since this is one of the most controversial elements of OPR. If you are interested but not ready to fully commit to open identities, as suggested above, you could start small with a pilot and scale up. Alternatively, you could allow reviewers to opt in or out (as happens, for example, at *eLife* and *MDPI*). As said above, to signal the journal’s support for the concept but allow reviewers choice, you could make open identities the default but enable reviewers to opt out of the process. Another possibility would be to maintain a standard single- or double-blind review process but to publish reviewer names alongside the final article (the practice at the publisher Frontiers). Bear in mind, though, that changes in these conditions could introduce biases whereby reviewers who are inclined to be more lenient towards a manuscript may be more likely to accept the review.

### H) Open reports

**Fig. 1:**
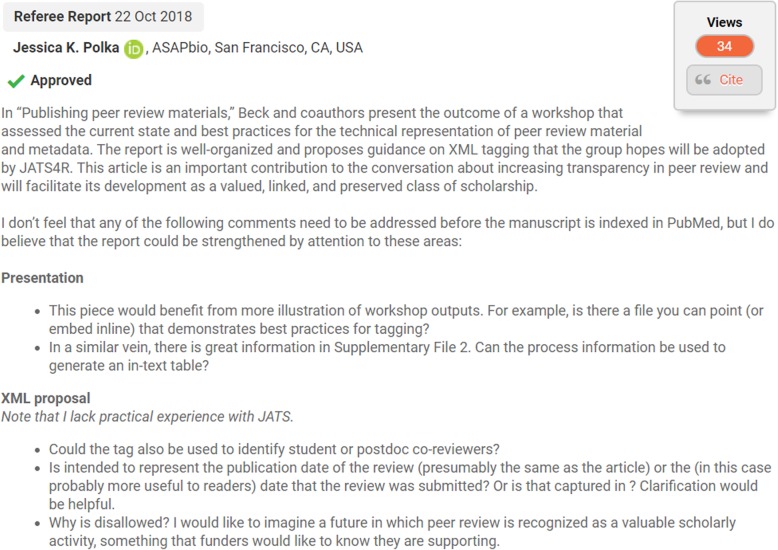
Screenshot of example published peer review report on *F1000Research* [22]

Open reports peer review is where review reports (either full reports or summaries) are published alongside the relevant article (an example is shown below in Fig. 1). Often, although not in all cases (e.g., *EMBO* reports), reviewer names are published alongside the reports. The main benefits of this measure lie in making currently invisible but potentially useful scholarly information available for re-use. There is increased transparency and accountability that comes with being able to examine normally behind-the-scenes discussions and processes of improvement and assessment and a potential to further incentivise peer reviewers by making their peer review work a more visible part of their scholarly activities (thus enabling reputational credit via linking reviews to researchers’ ORCID or Publons profiles, for example) (Fig. 1).

#### H1. Meet industry best-practice for publishing review reports

There is not yet an established industry standard for how to publish peer review reports, but this situation is now changing. The current best advice here is from Beck et al.’s 2018 article ‘Publishing peer review materials’, [20] whose first version (currently open for community review) advises that best practice is to assign individual DOIs to reports. In this way, review reports become a citable, discoverable and creditable part of the scholarly record in their own right. The authors see three routes to achieving the following:

1. ‘Peer review materials are attached to the article as a single or numerous PDFs. Whether these materials are pulled together into one document or attached as separate documents, there should be some defined mechanism in the JATS XML tagging that would support the capture of any available metadata and identify these files in a machine-readable and interoperable way for publishers to tag this content appropriately.
2. Peer review materials are appended to the article within the full text (so all is machine readable) as a sub-article component of the XML.
3. Peer review materials are full-text XML ‘articles’ or ‘commentaries’ in their own right that link bidirectionally to the main article.’

The options presented are in order from most basic to most complex, but also from least to most desirable. This means best practice would be option 3, but most pragmatic would be option 1. In addition, machine-readable metadata should accompany the content.

#### H2. Be aware of potential challenges in publishing reports

Beck et al. [20] advise that where publishers had existing workflows to prepare review content for publication, this could be done in minutes. Others advised they could spend 20 to 40 min for each article in tasks like removing ‘boilerplate text’ from reports, compiling content from multiple locations and editorial checks including reviewing/redacting sensitive information or inflammatory language. Clear policies should be in place for editors handling or overseeing any derogatory or defamatory remarks, and these should be publicly available on the peer review policy pages of the journal website. Where changes or redactions occur as a result in the published reports, a disclaimer could be added to indicate this is the case. It is also important to ensure coherent version management so reports can be linked to specific versions of manuscripts. A related issue here is the status of confidential reviewer messages to the editor—should these be allowed, will they be published later? If not, consider adding a further disclaimer to any published reports that these comments have been omitted. Finally, as this is an area in which there is not yet infrastructure to enable publishing of reports in a scalable and sustainable way, there may be substantial resources and cost commitments involved in publishing peer review information, which could be a significant barrier for smaller players (see the ‘Plan technologies and costs’ section for suggestions on how to deal with this).

### I) Open participation, pre-review manuscripts and open final version commenting

Open participation peer review allows the wider community to contribute to the review process. This can be either during publication (by making a pre-review manuscript openly available online as a pre-print or discussion paper) or after publication (by enabling comments on the publisher website or via a third-party platform like PubPeer^1^). Whereas in traditional review, editors identify and invite specific parties (peers) to review, open participation processes allow any interested members of the scholarly community or other interested parties from outside traditional scholarly circles to participate in the review process, either by contributing full, structured reviews or shorter comments. Often open participation processes will be used as a complement to traditional, invited reviews. Crowdsourcing reviewers in this way in theory ensures that fields do not become too insular or self-referential, enabling cross-disciplinary perspectives and potentially increase the number of researchers who can contribute to the quality assurance of manuscripts.

#### I1. Decide who can comment

**Fig. 2:**
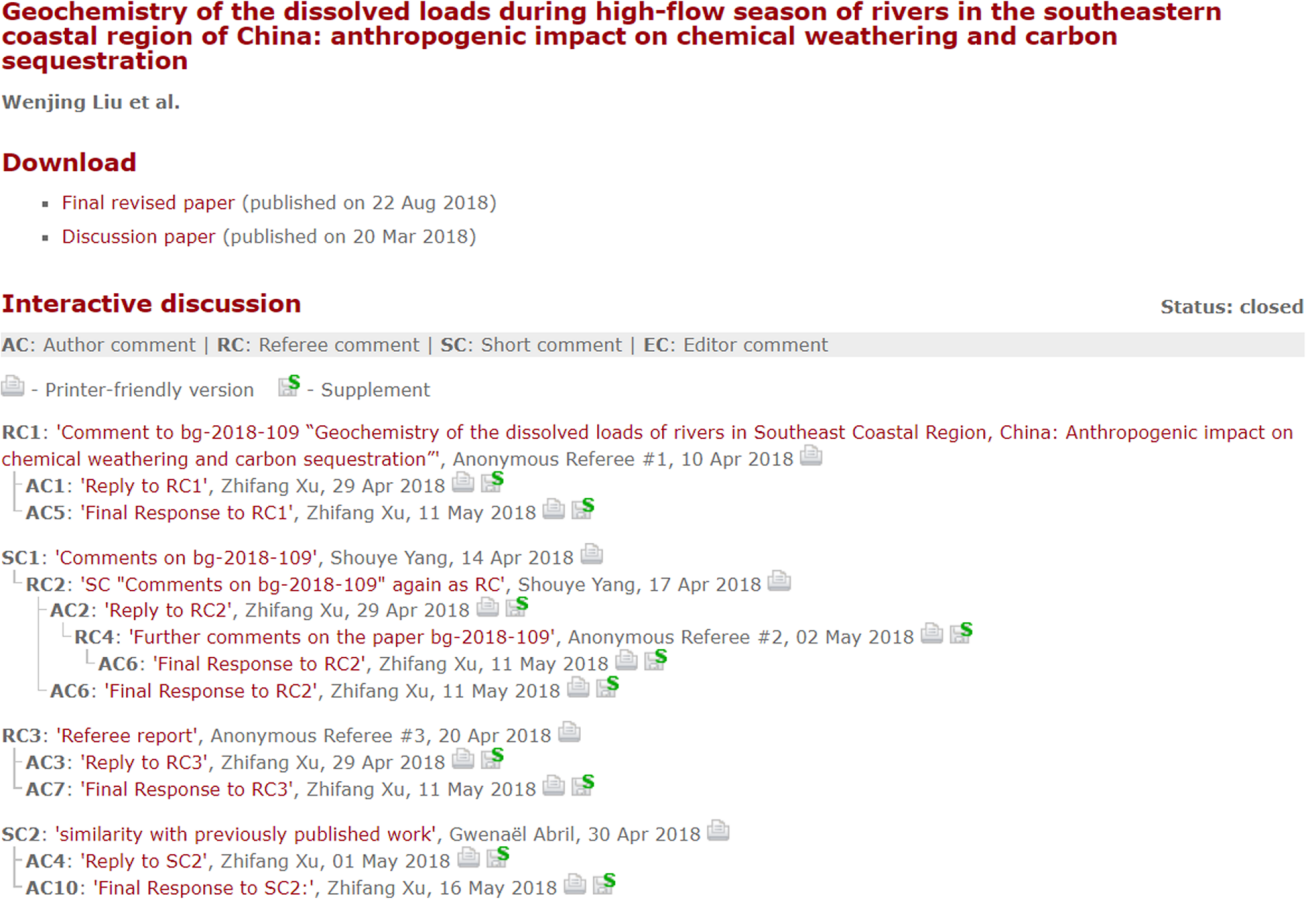
Screenshot of comment threads of open participation, pre-publication community discussion on an article in the *Copernicus* journal *Biogeosciences* https://www.biogeosciences.net/15/4955/2018/bg-15-4955-2018-discussion.html

A key decision here is whether to make comments open to anybody (anonymous or registered) or whether to require some credentials before allowing comments. Various options are available depending on your own communities. At *Copernicus Publications*, for example, reviewers can be anonymous but open commentators on discussion papers must add their identities [12] (Fig. 2). However, although MDPI’s *Preprints* service^2^ originally allowed only registered users to comment, this condition was recently relaxed. Despite concerns that this would lead to a lower-quality of comments, MDPI in fact reports having had few problems so far. One issue here is that for indexing services to accept comments (as, for example, *PubMed Central*^3^ accepts comments from *F1000Research*^4^), they may require a named individual and their affiliation.

#### I2. Consider how to foster uptake

A further crucial issue is that open participation processes often experience low uptake. Thus, as already said, they are hence often used as a complement to a parallel process of solicited peer review. At the open access journal *Atmospheric Chemistry and Physics* (ACP), which publishes pre-review discussion papers for community comments, only about one in five papers is commented upon [6]. Hence, open participation review is arguably better seen as a complement to, rather than a replacement for, invited peer review. In any case, some mediation of the community will help to stimulate engagement. Such mediation could take the form of reaching out to potential commentators directly to ask them to comment, or highlighting conversations via other channels such as social media, to entice others to engage.

### J) Open interaction

In traditional peer review, reviewers and authors correspond only with editors. Reviewers have no contact with other reviewers, and authors usually have no opportunity to directly question or respond to reviewers. Open interaction peer review allows and encourages direct reciprocal discussion between reviewers and/or between author(s) and reviewers.

#### J1. Decide which workflow to enable

Allowing interaction among reviewers or between authors and reviewers, or between reviewers themselves, is another way to ‘open up’ the review process, enabling editors and reviewers to work with authors to improve their manuscript. It is therefore important to decide what workflow you will follow. Examples of journals which enable pre-publication interaction between reviewers are the *EMBO Journal*^5^ and *eLife*.^6^ *Frontiers*^7^ has gone a step further, including an interactive collaboration stage with dialogue between authors, reviewers and editor(s).

#### J2. Be alert to how this may affect editorial workloads

While this extended dialogue might be expected to increase the editorial workload in some parts of the process, publishers practicing such methods actually report that they can also reduce workload in other parts. For example, the *eLife* consultation approach involves more work upfront (i.e., the consultation process and the drafting of the consensus decision letter), but time is saved later on if the editor decides on the revised version rather than sending back to the referees [21].

## Checklist

**Table:** 

Item	Guideline	Completed
General advice		
A) Set your open peer review goal(s)		
A1.	Decide what you’d like to achieve with OPR	
A2.	Acquaint yourself with the differences between the elements of OPR	
A3.	Decide which elements you would like to implement	
B) Listen to research communities		
B1.	Be conscious of, and sensitive to, community differences	
B2.	Consider surveying community opinions	
B3	Communicate your goal with the stakeholders and research community	
C) Plan technologies and costs		
C1.	Assess technological feasibility of various options	
C2.	Assess the costs of various options	
C3.	Consider work-around options for piloting	
D) Be pragmatic in your approach		
D1.	Set priorities and consider a phased approach	
D2.	Consider making options optional or piloting them first	
E) Further communicate the concept		
E1.	Engage the community, especially via “open champions”	
E2.	Be aware that communication is key and terminology is important	
F) Evaluate performance		
F1.	Have a clear framework for assessing success	
F2.	Accept that change takes time, but adjust if necessary	
F3.	Share your results with the community	
Trait-specific advice		
G) Open identities		
G1.	Devise strategies to compensate for the possibility that open identities might make it harder to find reviewers	
G2.	Be alert to possible negative interactions and have a workflow for dealing with them	
G3.	Enable credit	
G4.	Consider piloting or making open identities optional	
H) Open reports		
H1.	Meet industry best-practice for publishing review reports	
H2.	Be aware of potential challenges in publishing reports	
I) Open participation, pre-review manuscripts & open final version commenting		
I1.	Decide who can comment	
I2.	Consider how to foster uptake	
J) Open interaction		
J1.	Decide which workflow to enable	
J2.	Be alert to how this may affect editorial workloads	

## Conclusions

Open peer review is moving into the mainstream, but it is often poorly understood and surveys of researcher attitudes show important barriers to implementation. As more journals move to implement and experiment with the myriad of innovations covered by this term, there is a clear need for best practice guidelines to guide implementation. This article has aimed to address this knowledge gap, reporting work based on literature research, expert interviews and an interactive stakeholder workshop to create best-practice guidelines for editors and journals who wish to transition to OPR. The guidelines offer practical and pragmatic advice to these purposes at both a general level and for specific OPR traits. Main points of guidance are (a) set open peer review goal(s), (b) listen to research communities, (c) plan technologies and costs, (d) be pragmatic in approach, (e) further communicate the concept and (f) evaluate performance.

It is important to recognise some limitations of our approach to the creation of these guidelines, however. Firstly, our approach was to bring initial ideas forward individually and anonymously, but then to iteratively work on the development of the guidelines collaboratively with our whole group of experts. While we believe this open, collaborative phase enabled rich and in-depth discussion, it is possible that these group dynamics resulted in some ‘bandwagon effects’ whereby our experts influenced each other in their stated opinions. Secondly, these guidelines have not yet been pilot-tested, as would happen in the development of, for example, formal reporting guidelines. Nonetheless, given the range of experience and knowledge that has been incorporated here, we are confident the guidelines bespeak the best advice possible for the implementation of open peer review at this time. The area of open peer review is fast evolving. As journals and publishers look to experiment with new processes, it is our hope these guidelines prove useful in setting expectations and guiding best-practice.

## Additional file

Additional file 1: Background resources for open peer review implementation. (DOCX 24 kb)

## Abbreviation

OPR: open peer review

## Footnotes

1 https://pubpeer.com/

2 https://www.preprints.org/

3 https://www.ncbi.nlm.nih.gov/pmc/

4 https://f1000research.com/

5 http://emboj.embopress.org/

6 https://elifesciences.org/

7 https://www.frontiersin.org/
