# Supplementary material for: Guidelines for open peer review implementation
Source: Res Integr Peer Rev. 2019 Feb 27;4:4. doi: 10.1186/s41073-019-0063-9 (PMC6394088; doi:10.1186/s41073-019-0063-9)
Supplement: Supplementary file 1 — Background resources for open peer review implementation. (DOCX 24 kb) [file 41073_2019_63_MOESM1_ESM.docx]

Background resources for open peer review implementation

This is a suggested list of further reading for those aiming to implement open peer review at scholarly journals.

*2^nd^ Feb 2019*

# General advice

- Bravo G., Grimaldo F., López-Iñesta E., Mehmani B., & Squazzoni F. (2019). The effect of publishing peer review reports on referee behavior in five scholarly journals. Nature Communications 10(1):322 <http://doi.org/10.1038/s41467-018-08250-2>

# Bruce, R., Chauvin, A., Trinquart, L., Ravaud, P., & Boutron, I. (2016). Impact of interventions to improve the quality of peer review of biomedical journals: a systematic review and meta-analysis. BMC Medicine 14: 85. <https://doi.org/10.1186/s12916-016-0631-5>

- Glonti, K., Cauchi, D., Cobo, E., Boutron, I., Moher, D., & Hren, D. (2017). A scoping review protocol on the roles and tasks of peer reviewers in the manuscript review process in biomedical journals. BMJ Open. 7. <http://doi.org/10.1136/bmjopen-2017-017468>
- Groves, T. (2010). Is open peer review the fairest system? Yes. BMJ 2010, 341:c6424. <https://www.bmj.com/content/341/bmj.c6424.full>
- Hames, I. (2014). The peer review process: challenges and progress. Interview with Dr. Irene Hames. <https://www.editage.com/insights/the-peer-review-process-challenges-and-progress>
- Ross-Hellauer, T. (2017). What is open peer review? A systematic review [version 2; referees: 4 approved]. F1000Research 2017, 6:588 <https://doi.org/10.12688/f1000research.11369.2>
- Tennant, J., Dugan, J. M., Graziotin, D. et al. (2017). A multi-disciplinary perspective on emergent and future innovations in peer review [version 3; referees: 2 approved]. F1000Research, 6:1151. <https://doi.org/10.12688/f1000research.12037.3>
- Walker, R., & Rocha da Silva, P. (2015). Emerging trends in peer review-a survey. Frontiers in Neuroscience, 9, 169. <https://doi.org/10.3389/fnins.2015.00169>
- Wilsdon, J. et al. (2015). The Metric Tide: Report of the Independent Review of the Role of Metrics in Research Assessment and Management. DOI: 10.13140/RG.2.1.4929.1363. <https://responsiblemetrics.org/the-metric-tide/>
- Tennant, J. (2018). The state of the art in peer review. FEMS Microbiology Letters 365(19), fny204. <https://academic.oup.com/femsle/article/365/19/fny204/5078345>

# Researcher perspectives/attitudes

- Baggs J., Broome, M., Dougherty, M., Freda, M., & Kearney, M. (2008) Blinding in peer review: The preferences of reviewers for nursing journals. Journal of Advanced Nursing 64 (2), 131–138. <https://doi.org/10.1111/j.1365-2648.2008.04816.x>
- Kirkham J., & Moher, D. (2018). Who and why do researchers opt to publish in post-publication peer review platforms? - Findings from a review and survey of F1000 Research [version 1; referees: 2 approved, 1 approved with reservations]. F1000Research 7:920. <https://doi.org/10.12688/f1000research.15436.1>
- Pier, L. (2017). What is open peer review - and should I be doing it? Blog post. <https://libbypier.com/thoughts-musings/2017/7/14/what-is-open-peer-review>
- Publons. (2018). Global state of peer review. <https://doi.org/10.14322/publons.GSPR2018>
- Ross-Hellauer, T., Deppe, A., & Schmidt, B. (2017) Survey on open peer review: Attitudes and experience amongst editors, authors and reviewers. PLoS ONE 12(12). <https://doi.org/10.1371/journal.pone.0189311>
- Tattersall, A. (2015) For what it’s worth – the open peer review landscape. Online Information Review, 39 (5). 649 -663. <https://doi.org/10.1108/OIR-06-2015-0182>
- Ware, M. (2016). [Peer Review Survey 2015: Key Findings](http://publishingresearchconsortium.com/index.php/134-news-main-menu/prc-peer-review-survey-2015-key-findings/172-peer-review-survey-2015-key-findings) <http://publishingresearchconsortium.com/index.php/134-news-main-menu/prc-peer-review-survey-2015-key-findings/172-peer-review-survey-2015-key-findings>
- Wilkinson, J. (2017). Tracking Global Trends in Open Peer Review. Publons. <https://publons.com/blog/who-is-using-open-peer-review>/

# Open identities

- Bastian, H. (2017). The Fractured Logic of Blinded Peer Review. Bias Science Communication (PLoS Blogs). <https://blogs.plos.org/absolutely-maybe/2017/10/31/the-fractured-logic-of-blinded-peer-review-in-journals/>
- Budden, A., Tregenza, T., Aarsen, L. et al. (2008). Double-Blind Review Favours Increased Representation of Female Authors. Trends in Ecology & Evolution 23(1): 4–6. <https://doi.org/10.1016/j.tree.2007.07.008>
- Kowalczuk, M., & Samarasinghe, M. (2017). Comparison of Acceptance of Peer Reviewer Invitations by Peer Review Model: Open, Single-blind, and Double-blind Peer Review. Presented at the Peer Review Congress. <https://peerreviewcongress.org/prc17-0227>
- Ross, J., Gross, C., Desai, M. et al. (2006). Effect of Blinded Peer Review on Abstract Acceptance. Journal of the American Medical Association 295(14): 1675–80. <https://doi.org/10.1001/jama.295.14.1675>
- Tennant, J. (2017). The Dark Side of Peer Review. Editorial Office News 10 (8), 2-4. <https://doi.org/10.18243/eon/2017.10.8.1>
- Tomkins, A., Zhang, M., & Heavlin, W. (2017). Reviewer bias in single- versus double-blind peer review. PNAS 114 (48), 12708-12713. <https://doi.org/10.1073/pnas.1707323114>
- van Rooyen, S., Delamothe, T., & Evans, S. (2010). Effect on peer review of telling reviewers that their signed reviews might be posted on the web: randomised controlled trial. The BMJ 341. <https://doi.org/10.1136/bmj.c5729>
- van Rooyen, S., Godlee, F., Evans, S., Black, N., & Smith, R. (1999). Effect of open peer review on quality of reviews and on reviewers’ recommendations: a randomised trial. BMJ 318, 23–27. <https://doi.org/10.1136/bmj.318.7175.23>
- Walsh, E., Rooney, M., Appleby, L., & Wilkinson, G. (2000). Open peer review: A randomised controlled trial. British Journal of Psychiatry, 176(1), 47-51. <https://doi.org/10.1192/bjp.176.1.47>
- Yoder, J. (2014). Why we don’t sign our peer reviews. The Molecular Biologist. <http://www.molecularecologist.com/2014/04/why-we-dont-sign/>

# Open reports

- ASAPbio. (2018). FAQ on publishing peer review. <http://asapbio.org/pr-faq>
- Beck, J., Funk, K., Harrison, M. et al. (2018). Publishing peer review materials. F1000Research, 7:1655. <https://doi.org/10.12688/f1000research.16460.1>
- Cheeseman, I. (2018). Advocating for publishing peer review. <http://asapbio.org/cheeseman-advocating>
- Cosgrove, A., & Cheifet, B. (2018). Transparent peer review trial: the results. Genome Biology 19: 206. <https://doi.org/10.1186/s13059-018-1584-0>
- Hendricks, G., & Lin, J. (2017). Making peer reviews citable, discoverable, and creditable. Crossref. <https://www.crossref.org/blog/making-peer-reviews-citable-discoverable-and-creditable/>
- Hurst, P. (2018). Publication of peer review reports. The Royal Society. <https://blogs.royalsociety.org/publishing/publication-of-peer-review-reports/>
- Johnston, D. (2015). Peer review incentives: a simple idea to encourage fast and effective peer review. European Science Editing 41 (3), 70-71. <https://publons.com/blog/peer-review-incentives-a-simple-idea-to-encourage-fast-and-effective-peer-review/>
- Nature Communications. (2016). Transparent peer review one year on. Editorial. Nature Communications, 7, Article no. 13626. <https://doi.org/10.1038/ncomms13626>
- Polka, J., & Vale, R. (2018). It’s time to open the black box of peer review. ASAPbio. <https://asapbio.org/black-box>
- Polka, J., Kiley, R., Konforti, B., Stern, B.,& Vale, R. (2018). Publish peer reviews. Nature 560, 545-548. <https://doi.org/10.1038/d41586-018-06032-w>

# Open participation, pre-review manuscripts & open final version commenting

- Amsen, A. (2014). What is post-publication peer review? - F1000 Blogs. <https://blog.f1000.com/2014/07/08/what-is-post-publication-peer-review/>
- Bornmann, L., Herich, H., Joos, H., & Daniel, H.-D. (2012). In Public Peer Review of Submitted Manuscripts, How Do Reviewer Comments Differ from Comments Written by Interested Members of the Scientific Community? A Content Analysis of Comments.r Atmospheric Chemistry and Physics. Scientometrics 93(3), 915–29. <https://doi.org/10.1007/s11192-012-0731-8>
- Bourne, P., Polka, J., Vale, R., & Kiley, R. (2017). Ten simple rules to consider regarding preprint submission. PLoS Computational Biology 13(5). <https://doi.org/10.1371/journal.pcbi.10054733>
- Dolgin, E. (2018). PubMed Commons closes its doors to comments The US National Institutes of Health shutters its journal-commenting platform. Nature News. 02 February 2018. <https://doi.org/10.1038/d41586-018-01591-4>
- Pöschl, U. (2012). Multi-Stage Open Peer Review: Scientific Evaluation Integrating the Strengths of Traditional Peer Review with the Virtues of Transparency and Self-Regulation. Frontiers of Computational Neuroscience 6. <https://doi.org/10.3389/fncom.2012.00033>
- Ross-Hellauer, T. (2016). Disambiguating post-publication peer review. OpenAIRE Blog. <https://blogs.openaire.eu/?p=1205>
- Tennant, J. (2017). What are the barriers to post-publication peer review? Impact of Social Sciences. <http://blogs.lse.ac.uk/impactofsocialsciences/2017/04/12/what-are-the-barriers-to-post-publication-peer-review/>

# Open interaction

- [Kwon, J.](https://www.ncbi.nlm.nih.gov/pubmed/?term=Kwon%20JY%5BAuthor%5D&cauthor=true&cauthor_uid=28949810), Bulk, L., Giannone, Z., Liva, S., Chakraborty, B., & Brown, H. (2018). Collaborative peer review process as an informal interprofessional learning tool: Findings from an exploratory study. [Journal of Interprofessional Care](https://www.ncbi.nlm.nih.gov/pubmed/28949810) 32(1), 101-103. <https://doi.org/10.1080/13561820.2017.1358156>
- Zearfoss, R. (2017). How I learned to stop worrying about peer review and love collaboration. Crosstalk. <http://crosstalk.cell.com/blog/how-i-learned-to-stop-worrying-about-peer-review-and-love-collaboration>
- Pöschl, U., & Koop, T. (2008). Interactive open access publishing and collaborative peer review for improved scientific communication and quality assurance. Information Services & Use 28, 105–107. <https://doi.org/10.3233/ISU-2008-0567>
- King, S. (2017). Peer Review: Consultative review is worth the wait. <https://doi.org/10.7554/eLife.32012>

*The following contributed to this list of resources: Edit Gorogh, Iratxe Puebla, Jon Tennant, Jessica Polka, Stefan Reichmann, Tony Ross-Hellauer*
